# Vimentin Inhibits Dengue Virus Type 2 Invasion of the Blood-Brain Barrier

**DOI:** 10.3389/fcimb.2022.868407

**Published:** 2022-03-30

**Authors:** Jianhai Yu, Xujuan Li, Dongrui Zhou, Xuling Liu, Xiaoen He, Sheng-He Huang, Qinghua Wu, Li Zhu, Linzhong Yu, Jinxiu Yao, Bao Zhang, Wei Zhao

**Affiliations:** ^1^ Biological Safety Laboratory of Level 3 (BSL-3) Laboratory (Guangdong), Guangdong Provincial Key Laboratory of Tropical Disease Research, School of Public Health, Southern Medical University, Guangzhou, China; ^2^ Saban Research Institute of Children’s Hospital Los Angeles, Department of Pediatrics, University of Southern California, Los Angeles, CA, United States; ^3^ Department of Traditional Chinese Medicine, Southern Medical University, Guangzhou, China; ^4^ Department of Laboratory, People's Hospital of Yangjiang, Yangjiang, China

**Keywords:** dengue virus, vimentin, inhibition, human brain microvascular endothelial cells, SV129 mice

## Abstract

Dengue virus (DENV) causes dengue fever, which is prevalent in the tropical and subtropical regions, and in recent years, has resulted in several major epidemics. Vimentin, a cytoskeletal component involved in DENV infection, is significantly reorganized during infection. However, the mechanism underlying the association between DENV infection and vimentin is still poorly understood. We generated vimentin-knockout (Vim-KO) human brain microvascular endothelial cells (HBMECs) and a Vim-KO SV129 suckling mouse model, combining the dynamic vimentin changes observed *in vitro* and differences in disease course *in vivo*, to clarify the role of vimentin in DENV-2 infection. We found that the phosphorylation and solubility of vimentin changed dynamically during DENV-2 infection of HBMECs, suggesting the regulation of vimentin by DENV-2 infection. The similar trends observed in the phosphorylation and solubility of vimentin showed that these characteristics are related. Compared with that in control cells, the DENV-2 viral load was significantly increased in Vim-KO HBMECs, and after DENV-2 infection, Vim-KO SV129 mice displayed more severe disease signs than wild-type SV129 mice, as well as higher viral loads in their serum and brain tissue, demonstrating that vimentin can inhibit DENV-2 infection. Moreover, Vim-KO SV129 mice had more disordered cerebral cortical nerve cells, confirming that Vim-KO mice were more susceptible to DENV-2 infection, which causes severe brain damage. The findings of our study help clarify the mechanism by which vimentin inhibits DENV-2 infection and provides guidance for antiviral treatment strategies for DENV infections.

## Introduction

Dengue virus (DENV) is an important arbovirus with four serotypes, belongs to the family Flaviviridae, and causes dengue fever. More than 3 billion people are at risk of contracting DENV, and approximately 200 million people are infected each year (Artpradit et al., 2013; Yu et al., 2019). Unfortunately, there is no effective vaccine against dengue fever at present, and only symptomatic and supportive therapies exist (Farrar et al., 2007). It is crucial to develop novel host-targeted antiviral treatment strategies to avoid the development of viral resistance observed with direct-acting antiviral medications.

Several cytoskeletal proteins participate in viral life cycles. The cytoskeleton is a dynamic structure composed of three filament systems, namely microtubules, microfilaments, and intermediate filaments (IFs). Vimentin is a type III IF protein that is abundantly and widely expressed in eukaryotic cells (Satelli and Li, 2011). Not only does it contribute to cell adhesion, migration, organelle localization, and wound healing, but it is also involved in the infection process of several pathogens, such as African swine fever virus (Stefanovic et al., 2005), bluetongue virus (Bhattacharya et al., 2007), foot-and-mouth disease virus (Gladue et al., 2013), Japanese encephalitis virus (Liang et al., 2011), Parasites (He et al., 2017; Liu et al., 2022), Dengue virus, and SARS-CoV-2 (Ramos et al., 2020; Thalla et al., 2021).

Several studies have examined the specific role of vimentin in DENV infection. Surface vimentin on DENV-2-infected vascular endothelial cells (VECs) is highly colocalized with DENV-2 and directly interacts with it (Yang et al., 2016). DENV-2 infection results in vimentin fiber rearrangement in human umbilical vein endothelial cells and increases vimentin Ser71 phosphorylation (Bauer et al., 2012). Vimentin phosphorylation regulates many of its biological functions, including its roles in cell adhesion, migration, and signal transduction (Li et al., 2016; Gelens and Saurin, 2018; Zhang et al., 2020). Free soluble vimentin molecules exist in intracellular regions as precursors that can be assembled into insoluble filaments, and the ratio of soluble/insoluble vimentin is inversely proportional to the ease of filament rearrangement (Cogli et al., 2013; Wen et al., 2020). Different protein kinases phosphorylate filamentous vimentin at several sites to induce its disassembly, and increased phosphorylation is associated with vimentin rearrangement *in vitro* (Inagaki et al., 1987). The regulation of vimentin phosphorylation might also be involved in mediating the dynamic depolymerization of IFs through the regulation of vimentin solubility (Sripathi et al., 2012; Sjöqvist et al., 2021), which could alter its function (Pérez-Sala et al., 2015; Usman et al., 2021). However, the phosphorylation and solubility of vimentin after DENV infection have not been examined.

A possible pathogenic mechanism for neurological complications caused by dengue fever is direct invasion of the central nervous system (CNS) (Kumar and Margekar, 2016; Calderón-Peláez et al., 2019). The blood-brain barrier (BBB), composed of human brain microvascular endothelial cells (HBMECs), separates the blood and the CNS (Meena et al., 2021). Vimentin is involved in the viral life cycle as an IF to synthesize the cytoskeleton and inhibits dengue virus infection. Therefore, studies on vimentin phosphorylation could help to overcome the limitation of traditional drugs and treat dengue fever through another mechanism. In this study, HBMECs and an intracranial challenge mouse model, with or without vimentin knockout, were used to clarify the correlation between vimentin and DENV-2 infection *in vitro* and *in vivo*. These results suggest a possible host-targeted antiviral strategy to combat DENV infection, avoiding the risk of resistance inherent to the use of direct-acting antivirals.

## Materials and Methods

### Ethics Statement

Animal experiments were approved by the Ethical Committee for Animal Research of Southern Medical University and conducted based on the guidelines of the Ministry of Science and Technology of China.

### Viruses, Animals, and Cell Lines

The DENV-2 New Guinea C (NGC) strain (GenBank: KM204118.1) was obtained from our laboratory (Huang et al., 2016), propagated using mosquito C6/36 cells, and grown to 5×10^6^ PFU/mL. SV129 and SV129 (Vim-KO) mice were donated by Professor Sheng-he Huang (Los Angeles Children’s Hospital, University of Southern California, Los Angeles, CA, USA) (Huang et al., 2016a). Control (Con) and vimentin-knockout (Vim-KO) HBMECs were provided by Professor Bao Zhang (Southern Medical University, Guangzhou, Guangdong, China) and cultured in RPMI 1640 medium (Gibco, Shanghai, China) containing 10% fetal bovine serum (FBS, Gibco, Shanghai, China) (Zhu et al., 2019).

### Confocal Immunofluorescence Assay

HBMECs were plated in confocal culture dishes and grown to 80% confluence. After viral infection, the cells were fixed with 4% paraformaldehyde at 4°C for 30 min, permeabilized with 0.1% triton X-100 at 25°C for 20 min, blocked with 10% FBS at 37°C for 2 h, and incubated with primary antibody (mouse anti-vimentin monoclonal antibody, ab8978, Abcam) overnight at 4°C, followed by secondary antibody (TRITC, ab7065, Abcam, Shanghai, China) at 37°C for 1 h. After washing, the nuclei were stained with 4’,6-diamidino-2-phenylindole (BestBio, Shanghai, China) and mounted with an antifluorescent quencher (Panera AAPR11, Pythonbio, Guangzhou, China). The samples were analyzed on a confocal laser scanning microscope (FV1000-EVA, Olympus, Beijing, China).

### Real-Time Cellular Analysis

HBMECs (Con and Vim-KO) were plated in 8-well plates (E-Plate L8, ACEA Biosciences, San Diego, California, USA) for examination using an iCELLigence Label-Free Real-Time Cell Analysis System (ACEA Biosciences, San Diego, California, USA). The HBMECs were infected with DENV-2 at an multiplicity of infection (MOI) = 1 for 2 h and then cultured for 72 h. A control group infected with PBS was set up for each HBMEC experiment. The system collected data every 1 min and 10 min, respectively, during the infection and culture progress. In the data analysis system, each curve was normalized with the first Cell Index collected during infection or culture as the starting point (normalized cell index = 1), following which, a curve was generated based on the difference between the infected group and the control group to obtain the dynamic changes during DENV-2 infection or culture. Finally, the dynamic curves of HBMECs (Con and Vim-KO) were normalized using Min-Max Normalization with the formula x = (x − Min)/(Max − Min).

### Western Blot Analysis

HBMECs were cultured in 6-well plates and infected with DENV-2 (MOI = 1) for 0, 1, 2, 6, 12, 24, 36, and 48 h. To measure vimentin levels, total proteins were extracted with radioimmunoprecipitation buffer containing phenylmethylsulfonyl fluoride (PMSF, 8553S, Cell Signaling Technologies, Danvers, Massachusetts, USA) as a protease inhibitor. To measure vimentin phosphorylation levels, we used the ProteinExt^®^ Mammalian Total Protein Extraction Kit (Transgen Biotech, Beijing, China) supplemented with PMSF and phosphatase inhibitors (Roche PhosSTOP, 05892791001, Solarbio, Shanghai, China). Lysate protein concentrations were determined by a bicinchoninic acid assay. Proteins (60 μg) were resolved by sodium dodecyl sulfate-polyacrylamide gel electrophoresis (SDS-PAGE), incubated with primary antibody (Anti-Phospho-(Ser/Thr) Phe antibody, ab17464, Abcam, Shanghai, China) followed by secondary antibody (HRP-conjugated Affinipure Goat Anti-Mouse IgG(H+L), Abcam, Shanghai, China), and visualized by electrochemiluminescence (Biodlight Western Chemiluminescent HRP Substrate, BLH01S020, Bioworld Technology, Jiangsu, China).

To measure vimentin solubility levels, total proteins were extracted with lysis buffer containing 0.1 mM sodium orthovanadate (AAPR593, Pythonbio, Guangzhou, China). After centrifugation at 20,000 × *g* for 30 min at 4°C, samples were separated into supernatants and precipitates. The supernatants contained soluble vimentin, and the precipitates could be used to measure insoluble vimentin after dissolving in PBS (He et al., 2017). Protein concentrations were determined, and 60 μg of each sample was resolved by SDS-PAGE, incubated with primary antibody (mouse anti-vimentin monoclonal antibody) followed by secondary antibody (HRP-conjugated Affinipure Goat Anti-Mouse IgG(H+L)), and visualized by electrochemiluminescence (Biodlight Western Chemiluminescent HRP Substrate). Finally, vimentin solubility was calculated as follows: vimentin solubility = soluble/(soluble + insoluble) × 100%. Using β-actin mouse monoclonal antibody (3700S, Cell Signaling Technologies, Danvers, Massachusetts, United States of America) as a loading control, protein levels were quantified, and the proportion of soluble vimentin to total vimentin was calculated at each time point using ImageJ (version 1.51j8, National Institutes of Health, Bethesda, Maryland, USA).

### DENV-2 Infection of Suckling Mice

Homozygous female and male SV129 or SV129 (Vim-KO) mice were housed separately. Male and female mice born in the same litter were selected, and when they reached 9 weeks of age, they were bred together at a 1:1 ratio of males and females. The suckling mice were weighed immediately after birth, and those for which the body weight was outside the overall 25%–75% confidence interval was euthanized, whereas the others were randomly assigned to experiment groups. One-day-old SV129 and SV129 (Vim-KO) suckling mice (*n* = 6/group) were intracranially injected with 20 μL of DENV-2 at a titer of 2.6 × 10^6^ PFU/mL. The mice were observed for disease signs and weighed daily. On days 3, 4, and 5 after DENV-2 injection, one mouse in each group was euthanized to extract brain tissue and collect serum. The experiment was repeated in triplicate.

### DENV-2 Viral Load Detection

Cell culture, brain, and serum RNA was extracted using the QIAamp Viral RNA Mini Kit (Qiagen, Hilden, Germany) and reverse transcribed using the PrimeScript RT Reagent Kit (Takara Bio, Kusatsu, Japan), followed by qRT-PCR using Bestar^®^ Taqman qPCR Master Mix (Takara Bio). The DENV-2 plasmid was used as a standard to calculate viral copy numbers (in copies/g and copies/mL).

### Histopathology

Five days after intracranial DENV-2 injection in SV129 and SV129 Vim-KO mice, the brain tissues of three randomly selected mice per group were harvested and immediately fixed for 16–24 h in 10% neutral buffered formalin. Mice in the control groups were subjected to double-blind pathological examination. Tissues were submitted to Guangzhou Huayin Medical Science Company Limited (Guangzhou, China) for paraffin embedding; they were processed and sectioned at the same place before staining with hematoxylin and eosin and being subjected to microscopic examination for histopathological changes.

### Statistical Analysis

The averages of total expression data and solubility level data for vimentin in HBMEC cells after DENV-2 infection were analyzed by one-way analysis of variance with p = 0.05 and Dunnett-t tests. The averages of the Vim/actin ratio, DENV-2 titer, normalized cell index, daily weight, and viral load of cell culture, brain, and serum were analyzed by independent samples t-tests with p = 0.05 for each day and each group.

## Results

### DENV-2 Infection of HBMECs Causes Vimentin Rearrangement

**Figure 1:**
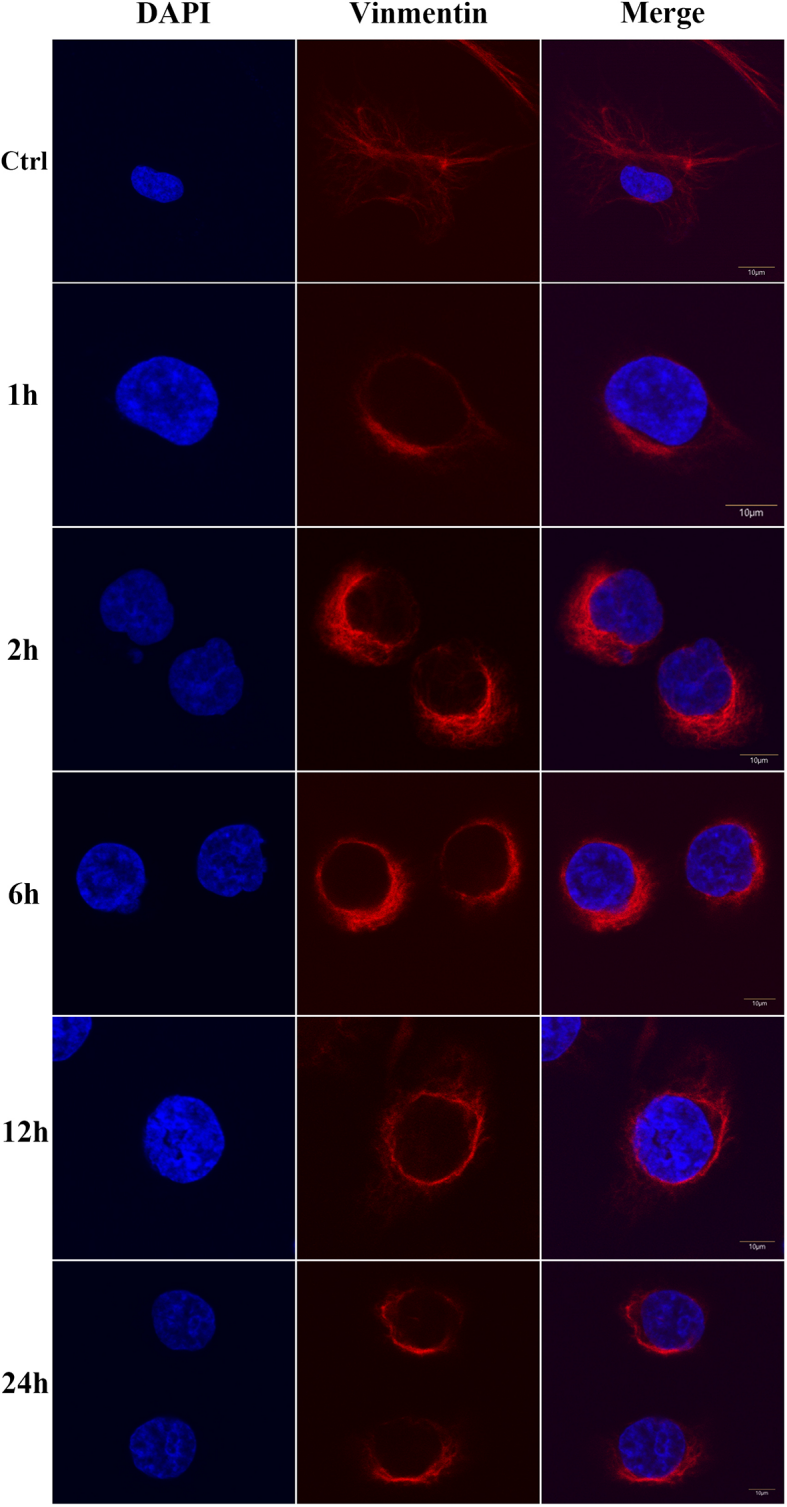
Vimentin rearrangement in human brain microvascular endothelial cells (HBMEC) infected with dengue virus (DENV)-2 for different periods of time. Ctrl means control group. All scale bars are 10 μm.

To explore how vimentin responds to DENV-2 infection at the cellular level, we examined HBMECs at different time points after DENV-2 infection using vimentin immunofluorescence. Vimentin was observed to be widely distributed in the cytoplasm in normal HBMECs but began to accumulate around the nucleus after 1 h of DENV-2 infection (**Figure 1**).

### Dynamic Changes in Vimentin Expression, Phosphorylation, and Solubility After DENV-2 Infection

**Figure 2:**
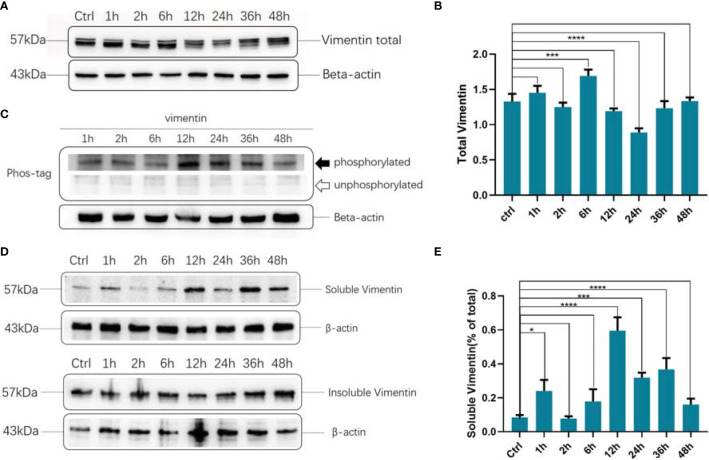
Dynamic changes in total protein, phosphorylation, and solubility of vimentin after dengue virus (DENV)-2 infection. **(A)** Changes in vimentin expression after DENV-2 infection of human brain microvascular endothelial cells (HBMEC) at different time points. **(B)** Grayscale analysis of the results of Figure **(A, C)** Changes in vimentin phosphorylation after DENV-2 infection in HBMECs were detected. **(D)** Soluble and insoluble fraction of vimentin at different time points of DENV-2 infection in HBMECs, as detected by western blotting. **E** Grayscale analysis of Figure **(D)**. **(A-E)** Ctrl: control group. **(B, E)** *P < 0.05; ***P < 0.001; ****P < 0.0001.

Total vimentin levels, phosphorylation, and solubility fluctuated at different time points after infection. Total vimentin levels decreased obviously 24 h after infection (**Figures 2A, B**). Vimentin phosphorylation peaked 12 h after infection and subsequently decreased (**Figure 2C**). Vimentin solubility was also highest 12 h after infection (**Figures 2D, E**).

### Vimentin Inhibits DENV-2 Invasion of HBMECs

**Figure 3:**
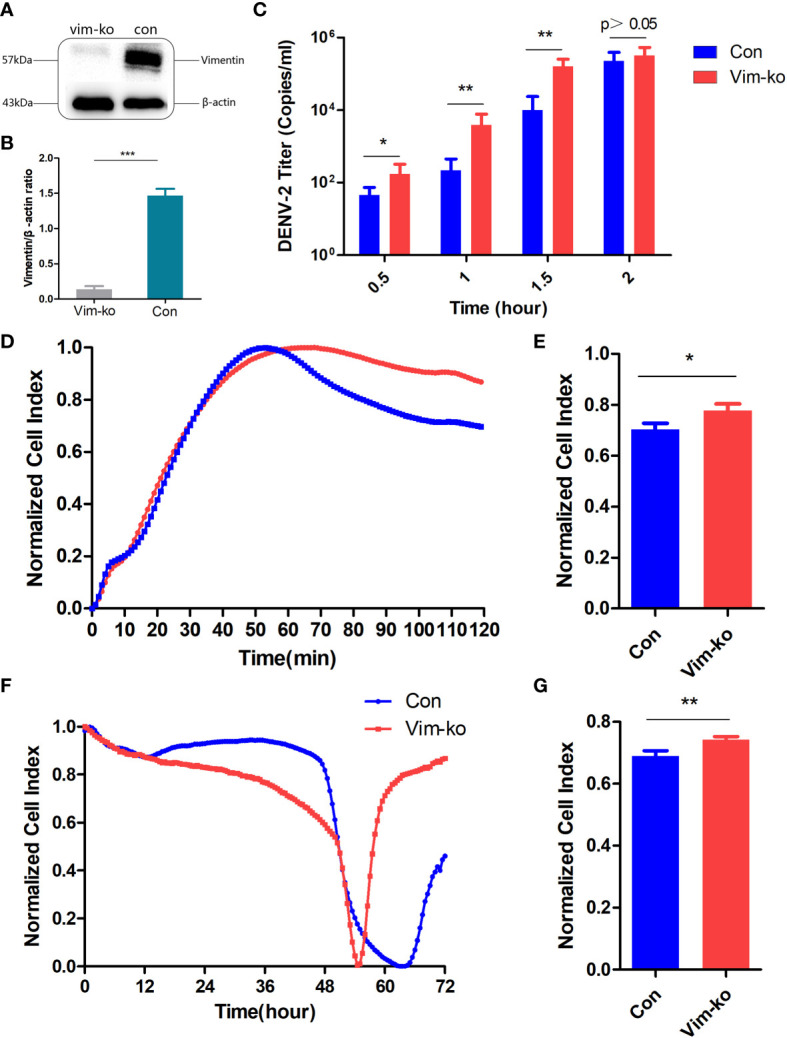
Vimentin might inhibit dengue virus (DENV)-2 invasion of human brain microvascular endothelial cells (HBMECs). **(A)** Vimentin expression in HBMECs (Con and Vim-KO). **(B)** Gray scale analysis of Figure **(A, C)** DENV-2 titers of intracellular virus in HBMECs (Con and Vim-KO). **(D, E)** Dynamic curves and normalized cell index of DENV-2 infected HBMECs (Con and Vim-KO) within 2h. **(F, G)** Dynamic curves and normalized cell index of DENV-2 cultured with HBMECs (Con and Vim-KO) within 72h. **(B, C, E, G)** *P < 0.05; **P < 0.01; ***P < 0.001.

Between the control (Con) and Vim-KO HBMEC lines, western blotting was used to verify the differences in vimentin expression levels and qRT-PCR was used to determine the differences in intracellular viral loads. Vimentin expression was significantly lower in Vim-KO HBMECs (**Figures 3A, B**). The rate of DENV2 infection into Vim-KO HBMECs was significantly faster than that with Con HBMECs (**Figure 3C**).

The ICELLigence Label-Free Real-Time Cell Analysis System was used to monitor biological state changes in cells. During DENV-2 infection, the cell resistance value of Vim-KO HBMECs was significantly higher than that of Con HBMECs, suggesting that Vim-KO HBMECs have higher cell permeability and are more conducive to virus invasion (**Figures 3D, E**). Similarly, in the process of DENV-2 culture, Vim-KO HBMECs reached the cytopathic effect endpoint (normalized cell index = 0) earlier than Con HBMECs, which means that Vim-KO HBEMCs were more conducive to virus replication and proliferation, causing cells to die faster due to viral load bursts (**Figures 3F, G**).

### Establishment of DENV-2-Infected Suckling Mouse Model

To verify the relationship between vimentin and DENV-2 infection *in vivo*, we generated mouse models of intracranial challenge, with and without vimentin knockout, and observed differences in disease course, signs, mortality, and body weight. The disease course could be divided into three distinct periods. Like that in the control mice, neither infected group displayed disease signs within 2 d of intracranial DENV-2 injection, indicating that this was the incubation period. Signs developed to different degrees in both infected groups on days 3 and 4 after intracranial injection but not in the uninfected control group; this was considered the onset period. Both groups of infected mice died within 6 d, with mortality rates as high as 100%; this was considered the death period.

**Figure 4:**
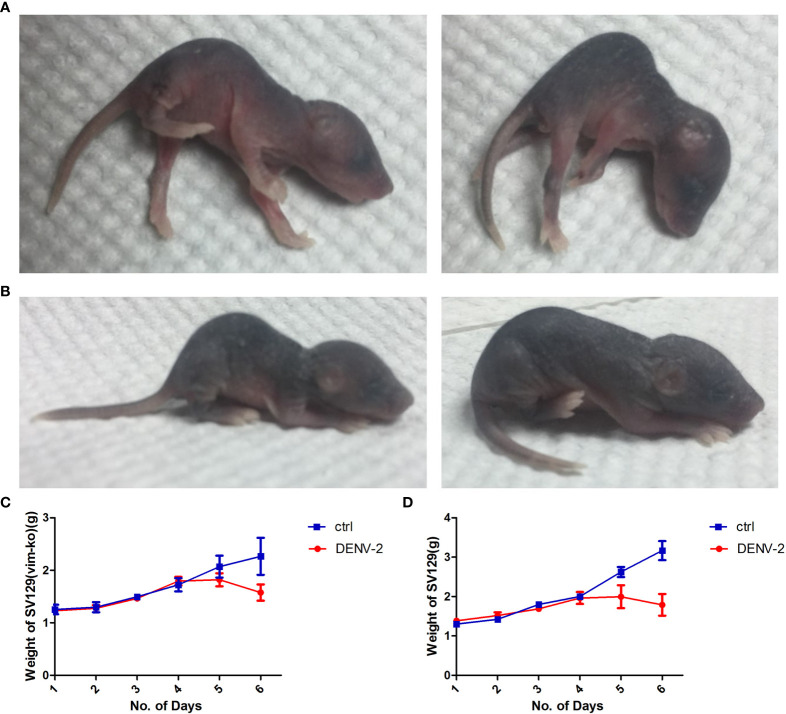
Disease signs and weight changes in SV129 and SV129 (Vim-KO) mice after infection with dengue virus (DENV)-2. **(A, B)** Signs in SV129 and SV129 (Vim-KO) mice with DENV-2 infection. **(C, D)** Weight changes in SV129 and SV129 (Vim-KO) mice with DENV-2 infection.

During the onset period, both groups of infected mice displayed mild signs 3 d after injection, including arching and slow walking, and SV129 Vim-KO mice displayed slight tremors. By day 4, mice in both infected groups had arched backs and low movement, and their hind limbs trembled while lying down. By the evening of day 4, both infected groups displayed severe back signs. The hind limbs were paralyzed and when placed flat on the table, they fell to one side. Those with milder signs could crawl with their forelimbs, but their hind limbs trembled constantly. SV129 Vim-KO mice showed lower limb weakness and could not walk while maintaining a side squat. On the 5th day after injection, SV129 mice retained slight use of their forelimbs for crawling, whereas Vim-KO mice completely lost their exercise capacity and had side-lying paralysis. Mice in both groups typically died by day 6; however, the signs were more severe in SV129 Vim-KO mice than in SV129 mice, and death occurred more quickly (**Figures 4A, B**). The weights of mice in each group were recorded daily. Those in the infected groups increased steadily on days 0–3, plateaued on day 4, and were significantly decreased compared to those of untreated mice by day 5 (*p* < 0.05; **Figures 4C, D**).

### Vimentin Inhibits DENV-2 Infection in Suckling Mouse Model

**Figure 5:**
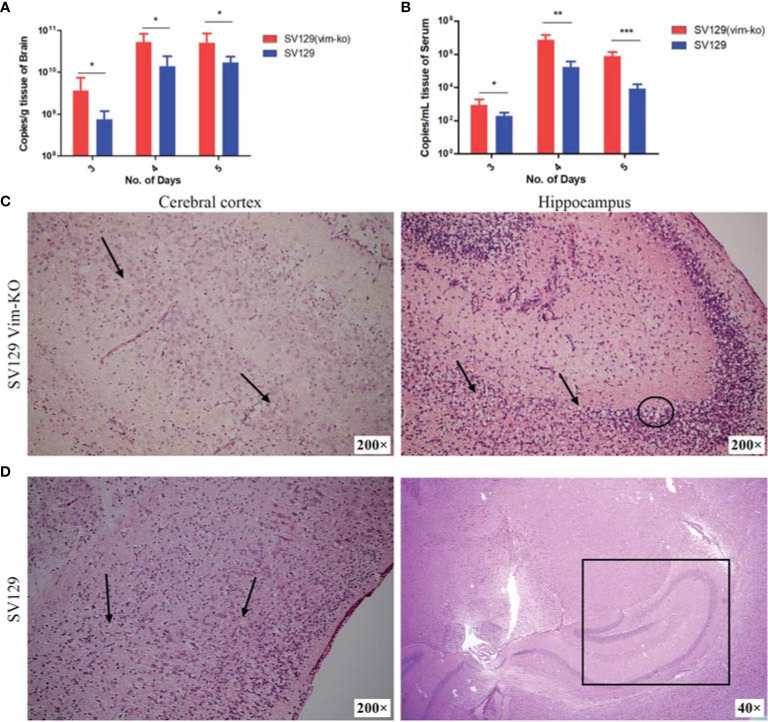
Detection of viral load in serum and brain and description of brain histopathological damage after infection of SV129 and SV129 (Vim-KO) mice with dengue virus (DENV)-2. **(A, B)** Changes in the viral load in the brain and serum; *P < 0.05; **P < 0.01; ***P < 0.001. **(C, D)** Brain histopathological section on the 5th day after infection in SV129 and SV129 (Vim-KO) mice. **(C)** Left: the cortical stratifications disappeared, with a large number of apoptotic pyknotic cells (black arrow). Right: the hippocampus displayed apoptotic pyknosis of glial cells (black arrow) and local cerebral liquefactive necrosis (black circled). **(D)** Left: the cortical stratification was normal, and apoptotic pyknosis of glial cells (black arrow) was found in all six cortical layers. Right: the hippocampus (black square) showed no obvious abnormalities.

The viral loads and brain histopathology also differed between the two infected groups. From day 3 after infection, we tested brain and serum samples for viral load and histopathology, since no significant signs or changes in body weight were observed and the brain tissue samples, and its circulating blood volumes were too small on the first 2 d after viral infection. In both SV129 Vim-KO and SV129 mice, the viral loads in brain and serum showed a trend of first increasing and then decreasing from day 3 to 5 after infection. However, regardless of which day or tissue, those in SV129 Vim-KO mice were significantly higher than those in SV129 mice (**Figures 5A, B**).

The histopathology of the brains of SV129 VIM-KO mice showed more severe damage, with not only disorganized nerve cells in their cerebral cortex, but also a large number of apoptotic pyknotic cells and neuronal damage; the cortical stratifications among the molecular layer, external granular layer, external pyramidal layer, internal granular layer, and internal pyramidal layer had disappeared. Moreover, the hippocampus even displayed apoptotic pyknosis of glial cells, edema, and local cerebral liquefactive necrosis (**Figure 5C**). In SV129 mice, although the neurons in the cerebral cortex were disordered with apoptotic pyknosis of glial cells, edema and spotty necrosis could also be found in all six cortical layers; the cortical stratification was normal, and the hippocampus showed no obvious abnormalities (**Figure 5D**).

## Discussion

In this study, we used HBMECs (to simulate the BBB) and a mouse model of intracranial challenge to explore the role of vimentin in DENV-2 invasion. The results suggested that targeting vimentin is a potential host-derived antiviral treatment strategy for DENV infection. At the cellular level, vimentin is involved in a variety of pathogen invasion and infection processes. It can promote *Escherichia coli* K1 invasion and increase BBB permeability and neuronal inflammation by regulating nuclear factor (NF)κB signaling to defend against meningitis (Huang et al., 2016a; Mak and Brüggemann, 2016). Vimentin also interacts directly with the spike protein of severe acute respiratory syndrome coronavirus during its invasion (Yu et al., 2016). The surface vimentin of DENV-2-infected VECs is highly colocalized with the virus, and the envelope domain III of DENV-2 directly interacts with the rod domain of surface vimentin to mediate DENV infection (Yang et al., 2016). Infection then induces vimentin rearrangement, which is closely related to Ser71 phosphorylation (Lei et al., 2013; Murray et al., 2014). In our study, morphological changes in DENV-2-infected HBMECs occurred within 1 h of infection, and vimentin went from being present throughout the cell to only being present around the nucleus. Marked re-localization of vimentin to the perinuclear region was observed, consistent with that in previous reports (Bauer et al., 2012; Cogli et al., 2013). In our study, both the phosphorylation and solubility of vimentin were highest at 12 h after viral infection, whereas the level of protein expression did not follow this trend, which indicates that vimentin expression, phosphorylation, and solubility are all regulated by DENV-2 infection.

The iCELLigence Label-Free Real-Time Cell Analysis System is a cellular monitor that uses electrical impedance sensors to continuously and quantitatively track the biological state of cells in real time (Şener et al., 2017; Stefanowicz-Hajduk and Ochocka, 2020). Vimentin is involved in the regulation of cell behavior, and the vimentin assembly state is sensitive to stimuli that alter cellular tension and morphology (Patteson et al., 2020). However, vimentin can have varying effects on pathogen invasion. For example, vimentin promotes infections by pathogens, such as *E. coli* K1 and Japanese encephalitis virus (Liang et al., 2011; Huang et al., 2016b) and inhibits the internalization of human papillomavirus type 16 pseudovirions into host cells (Schäfer et al., 2017). In our study, the adherent area of Con HBMECs increased at a lower rate than that of Vim-KO HBMECs, indicating that vimentin acts to maintain cellular morphology during DENV-2 invasion. In addition, after 2 h of infection, the viral load in Vim-KO HBMECs was higher than that in Con HBMECs, suggesting that vimentin inhibits DENV-2 invasion.

We verified the role of vimentin in DENV invasion in SV129 suckling mice with and without vimentin knockout. Many models have been used to study DENV, including A/J (Shresta et al., 2004), BALB/c (Ahmad et al., 2019), C57BL/6 (Byrne et al., 2020), and AG129 (Tan et al., 2010) mice for dengue virus tropism and pathogenic research; the DENV-infected non-human primate (Kayesh and Tsukiyama-Kohara, 2022), AG129 mouse continuous infection, cross-infection-established ADE mouse models to study the immune mechanisms of DENV infection (Blaney et al., 2002); and DENV-infected suckling mouse (Pelliccia et al., 2017) and DENV-infected SCID mouse models in vaccine research. In this study, we used suckling mice because adult mice displayed no obvious clinical signs after infection, and DENV-2 was not detected in the serum. This is consistent with previous studies, which suggested that the susceptibility of mice to DENV is inversely proportional to immune system maturity (Zellweger and Shresta, 2014; Ng et al., 2014; Mustafá et al., 2019). Subcutaneous injection resulted in low and unstable infection rates in both groups of suckling mice, preventing accurate comparisons between them. Intracranial injection was used because of the resulting reliable and stable infection levels and ease in observing neurological manifestations (Al-Shujairi et al., 2017).

Several studies have demonstrated the involvement of vimentin in the occurrence of pathogenic CNS infections in animal models, resulting in various signs and pathological changes. *Listeria monocytogenes* cannot colonize the brains of vimentin-knockout BALB/c mice (Ghosh et al., 2018). Vimentin knockout mice were also used to show that vimentin could enhance *E. coli* K1 penetration of the BBB through the NF-κB pathway, contributing to significant increases in α7 nicotinic acetylcholine receptor-mediated calcium signaling and neuronal injury (Huang et al., 2016a). After recombinant major capsid protein (VP1) was intracranially injected into C57BL/6J mice, increases in albumin and vimentin and decreases in tight junction proteins in brain tissues suggested that VP1 and vimentin might act in concert to increase BBB permeability and promote CNS infection (Wang et al., 2019). Vimentin serves as a surface receptor that binds enterovirus (EV)-A71 VP1, and SV129 Vim-KO mice display decreased body weight changes, morbidity, and cerebral cortex damage in comparison to those in SV129 control mice, indicating that vimentin knockout can reduce EV-A71 CNS infection *in vivo* (Zhu et al., 2019). After comparing the disease signs, body weights, intracranial, serum viral loads, and brain histopathology of the two groups of infected suckling mice, we observed that the signs in SV129 Vim-KO mice were more serious, with higher intracranial viral loads and serum viral loads on the 4th and 5th days after infection. The viral load in the brain tissue of 1 d-old SV129 Vim-KO mice after the intracranial injection of DENV-2 can be as high as 3.52 × 10^10^ copies/g, making them quite suitable for virus conservation and maintaining high viral titers. This study reveals for the first time that vimentin can inhibit the infection, replication, and release of DENV-2 in the brain tissue of suckling mice, and that it affects the viral load entering the circulation. In addition, the susceptibility of the SV129 Vim-KO model to intracranial DENV challenge makes it a useful tool for virulence assessments of live attenuated vaccines, other strains, RNA transcripts, and infectious clones, and it is also commonly used for virus preservation in our laboratory.

## Data Availability Statement

The original contributions presented in the study are included in the article/supplementary material. Further inquiries can be directed to the corresponding authors.

## Ethics Statement

The animal study was reviewed and approved by Ethical Committee for Animal Research of Southern Medical University.

## Author Contributions

JHY, XJL, DZ, and XLL conceived the project, designed the experiments, undertook experiments, and wrote the manuscript. XH performed the experiments and analyzed the data. SH provided mouse models, guided animal experiments, and analyzed the data. QW, LZ, LY, and JXY provided valuable structural insight and helped to write the manuscript. BZ participated in the construction and improvement of the research and guided the experimental process and article writing. WZ participated in the entire research process and provided financial support. All authors contributed to the article and approved the submitted version.

## Funding

This work was supported by the National Key R&D Program of China [grant number 2018YFC1602206]; the National Natural Science Foundation of China [grant numbers 31470271, 81730110, and 31670168]; the Yangjiang, Guangzhou, and Guangdong Science and Technology Program key projects [grant numbers 2019010, 201803040006, and 2021B1212030014]; and the Basic Research Project of Key Laboratory of Guangzhou [grant numbers 202102100001].

## Conflict of Interest

The authors declare that the research was conducted in the absence of any commercial or financial relationships that could be construed as a potential conflict of interest.

## Publisher’s Note

All claims expressed in this article are solely those of the authors and do not necessarily represent those of their affiliated organizations, or those of the publisher, the editors and the reviewers. Any product that may be evaluated in this article, or claim that may be made by its manufacturer, is not guaranteed or endorsed by the publisher.
